# Optimization of lithium content in LiFePO_4_ for superior electrochemical performance: the role of impurities†

**DOI:** 10.1039/c7ra10112k

**Published:** 2018-01-03

**Authors:** Kruti K. Halankar, B. P. Mandal, Manoj K. Jangid, A. Mukhopadhyay, Sher Singh Meena, R. Acharya, A. K. Tyagi

**Affiliations:** Chemistry Division, Bhabha Atomic Research Centre Mumbai-400085 India bpmandal@barc.gov.in aktyagi@barc.gov.in +91-22-25505151 +91-22-25592274; High Temperature and Energy Materials Laboratory, Department of Metallurgical Engineering and Materials Science, IIT Bombay Mumbai-400076 India; Solid State Physics Division, Bhabha Atomic Research Centre Mumbai-400085 India; Radiochemistry Division, Bhabha Atomic Research Centre Mumbai-400085 India; Homi Bhabha National Institute Mumbai-400085 India

## Abstract

Carbon coated Li_*x*_FePO_4_ samples with systematically varying Li-content (*x* = 1, 1.02, 1.05, 1.10) have been synthesized *via* a sol–gel route. The Li : Fe ratios for the as-synthesized samples is found to vary from ∼0.96 : 1 to 1.16 : 1 as determined by the proton induced gamma emission (PIGE) technique (for Li) and ICP-OES (for Fe). According to Mössbauer spectroscopy, sample Li_1.05_FePO_4_ has the highest content (*i.e.*, ∼91.5%) of the actual electroactive phase (*viz.*, crystalline LiFePO_4_), followed by samples Li_1.02_FePO_4_, Li_1.1_FePO_4_ and LiFePO_4_; with the remaining content being primarily Fe-containing impurities, including a conducting FeP phase in samples Li_1.02_FePO_4_ and Li_1.05_FePO_4_. Electrodes based on sample Li_1.05_FePO_4_ show the best electrochemical performance in all aspects, retaining ∼150 mA h g^−1^ after 100 charge/discharge cycles at C/2, followed by sample Li_1.02_FePO_4_ (∼140 mA h g^−1^), LiFePO_4_ (∼120 mA h g^−1^) and Li_1.10_FePO_4_ (∼115 mA h g^−1^). Furthermore, the electrodes based on sample Li_1.05_FePO_4_ retain ∼107 mA h g^−1^ even at a high current density of 5C. Impedance spectra indicate that electrodes based on sample Li_1.05_FePO_4_ possess the least charge transfer resistance, plausibly having influence from the compositional aspects. This low charge transfer resistance is partially responsible for the superior electrochemical behavior of that specific composition.

## Abstract 2

Extensive information regarding the Li : Fe stoichiometry in the LiFePO_4_ cathode and the formation of concomitant impurities and their impact on the various electrochemical performances have been reported.

## Graphical abstract

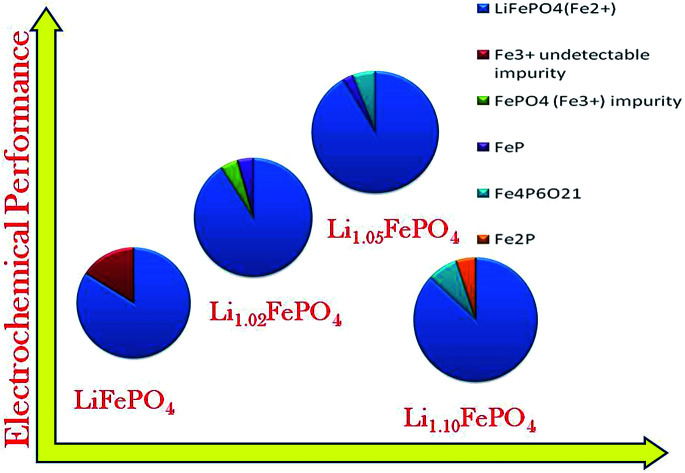

## 1. Introduction

Lithium iron phosphate (LiFePO_4_) has been extensively investigated as a safer and more environmentally friendly cathode material for Li-ion rechargeable batteries.^1^ This olivine structured cathode material also has an excellent theoretical specific capacity of 170 mA h g^−1^ and a flat charge–discharge profile at ∼3.4 V *vs.* Li/Li^+^ due to two-phase reaction.^1,2^ Being less expensive and safer than LiCoO_2_ makes it a better choice for large-scale applications such as hybrid and plug-in hybrid electric vehicles. However, LiFePO_4_ possess inherently poor electronic and ionic conductivities. Many approaches, such as reducing the particle size to nano sizes,^2–5^ coating with conductive carbon^6–10^ and doping LiFePO_4_ with various cations^11–13^ has been proposed to circumvent the same.

In order to obtain LiFePO_4_ with smaller particle size (preferably, in the nanosized regime, along with a conducting carbon coating), carbon sources like sucrose, glucose, fatty acids, polyaniline *etc.* have been used during synthesis by wet chemical methods.^14–21^ However, it has been frequently observed that during synthesis, in presence of above mentioned carbon sources, several impurity phases also form. Some of the impurities, like Fe_2_P and FeP, are believed to be useful, while others, such as Fe_4_P_6_O_21_ and Fe_2_P_2_O_7_ are detrimental towards the electrochemical performance of LiFePO_4_. The formation of these types of Li-deficient compounds is not surprising since energetically they have been reported to be more favourable.^22^ Formation of the Li-deficient compounds takes place due to volatility of lithium above 600 °C.^19^ Since Li-loss due to volatilization is unavoidable, researchers attempt using Li precursors in excess of those corresponding to the stoichiometric compositions. However, these are usually done fairly arbitrarily, with the excess lithium often precipitating as Li_3_PO_4_ which is electrochemically inactive and just add up to the dead weight. Also, these impurities, whether formed due to Li-deficit or Li-excess are often resistive in nature and thus highly undesirable.^23^

Accordingly, it is very important to determine very precisely the excess quantities of Li-precursors that need to be added to result in the optimum composition/stoichiometry, which need not be 100% phase pure LiFePO_4_, but which may lead to the best possible combination of electrochemical performances. For example, Hu *et al.*^24^ attempted to prepare non-stoichiomtric LiFePO_4_ by using precursors with Li : Fe in 1 : 2 ratio, which resulted in lower content of detrimental Li_3_PO_4_ and higher content of the desired Fe_2_P as the impurity phases. However, the content of LiFePO_4_ was low which led to lower specific capacity of the sample. There are few more reports on the effects of lithium non-stoichiometry on the electrochemical performances of LiFePO_4_, which also include discussions on how non-stoichiometry influences the particle size and lattice defects in LiFePO_4_.^25–27^

However, extensive information, especially based on systematic studies, regarding the Li : Fe stoichiometry attained, the concomitant impurity contents and their impact on the various electrochemical performances is scarcely reported in the literature. The challenge lies in precisely determining the lithium and iron concentrations in the as-synthesized samples. In most of the reported work, either atomic absorption spectroscopy (AAS) or inductively coupled plasma-optical emission spectroscopy (ICP-OES) has been used to determine the concentrations of Fe, as well as of Li.^26,28^ AAS and ICP-OES are not sensitive enough for determining the lithium concentration very precisely.

Accordingly, in the present work proton induced gamma emission (PIGE) technique has been used to determine the lithium concentration in the as-synthesized Li_*x*_FePO_4_-based samples. PIGE is an isotope specific nuclear analytical technique capable of determining elements with very low Z (such as Li, B, F, N, Si, Al) using low energy proton beam (2–5 MeV). It can determine concentrations of elements non-destructively in complex materials like ceramics, glass and carbides, which are otherwise difficult to be analysed using conventional wet-chemical methods. PIGE is a particularly sensitive method for Li, which involves measurement of prompt gamma-rays at 478 keV from ^7^Li (p, p′γ)^7^Li. On the other hand ^57^Fe Mössbauer spectroscopy is very sensitive to determine the Fe-based impurities and accordingly has been used for the same in present work.

PIGE and Mössbauer spectroscopy has been used simultaneously to determine the final Li : Fe atomic ratios including impurity phases. Their influences on the electrochemical performances have been investigated for a set of Li_*x*_FePO_4_-based samples synthesized with systematic variation of the starting Li contents (*i.e.*, precursor amounts). The impacts of Li-deficit, as well as Li-excess, on the phase/impurity contents and the concomitant electrochemical properties like charge transfer resistances, charge/discharge capacities, rate capabilities and cyclic stabilities have been discussed. Accordingly, the correlations between composition/stoichiometry, phase assemblage, impurity contents and electrochemical behavior as obtained for the first time in such systematically conducted study not only highlights the importance of precise control of the stoichiometry while synthesizing Li_*x*_FePO_4_-based electrode materials, but also shed light into the desired Li : Fe atomic ratio for obtaining the best possible electrochemical performances.

## 2. Experimental details

### 2.1. Materials synthesis

Li_*x*_FePO_4_-based samples with varying starting Li contents (*viz.*, *x* = 1.0, 1.02, 1.05, 1.10) were prepared by sol–gel method. The required amounts of CH_3_COOLi·2H_2_O (99.9%) and FeCl_2_·4H_2_O (99.9%) were first mixed in ethanol and stirred for 2 h, followed by addition of P_2_O_5_ (99.9%) and further stirring for 2 h. Subsequently, oleic acid was added to the solution as carbon source. The solution was stirred for another 3 h, followed by drying at 80 °C to form dry powder, which was then ground and annealed under reducing environment of H_2_ (8%) and Ar (92%) at 650 °C for 5 h. The carbon content of the samples was determined using CHNS analyser and was found to be ∼10 wt% in all the samples. The Li_*x*_FePO_4_ (or LFP) based samples with the starting Li-contents corresponding to *x* = 1.0, 1.02, 1.05 and 1.10 will hitherto be referred as sample A, B, C and D, respectively.

### 2.2. Phase assemblage and elemental analysis

In order to determine the crystal structure and phase purity, powder X-ray diffraction (XRD) was recorded for all the samples in the 2*θ* range of 10–70° using Rigaku SmartLab XRD unit having copper *K*_α_ source (and calibrated using silicon standard). The XRD profiles of the samples were refined using FullProf software. The particle and surface morphologies were studied using SEM (AIS 210, Mirero Inc., South Korea). Elemental mapping of the samples were done using EDAX attached with SEM instrument. Raman spectroscopy was performed using Horiba-Yvon instrument in the range 1000–1800 cm^−1^. The power of the laser was kept around ∼1 mW to avoid carbon burning at high power. Mössbauer spectra (MS) at room temperature were recorded with a conventional spectrometer operated in constant acceleration mode in transmission geometry with ^57^Co source in Rh matrix of 5 mCi. The recorded MS were fitted using the WinNormos fit program. The calibration of the velocity scale was done by using an enriched α-^57^Fe metal foil. The isomer shift values are relative to Fe metal foil (*δ* = 0.0 mm s^−1^).

The concentration of iron was measured using Inductively Coupled Plasma-Optical Emission Spectroscopy (ICP-OES). Li-content was precisely determined using proton induced gamma emission (PIGE) technique. An *in situ* current normalized PIGE method has been developed by us, which was used earlier for non-destructive determination of Li-content in Li-doped neodymium di-titanate and lithium titanate ceramics^29,30^ and boron in boron-based compounds, including B_4_C.^31^ Accordingly, the optimized PIGE method has now been used for determination of Li concentrations in the four LiFePO_4_ samples as developed here with varied starting Li-contents (*i.e.*, LiFePO_4_, Li_1.02_FePO_4_, Li_1.05_FePO_4_, Li_1.10_FePO_4_). As mentioned earlier, this is important because during synthesis Li has a tendency to sublime and also form compounds with Fe (*i.e.*, impurity phases). The four samples with varied Li concentrations were analysed by the PIGE method with fluoride (in the form CaF_2_) as *in situ* current normalizer. The concerned samples and lithium phosphate standards (75 mg each) were pelletized in cellulose as major matrix, with constant amount of fluoride (in the form of CaF_2_). The method was also validated by analysing lithium acetate and lithium carbonate samples. The samples and standard pellets were irradiated (in vacuum at 10^−6^ torr) using 4 MeV proton beam from Folded Tandem Ion Accelerator (FOTIA) at 15 nA current. Radioactive assay of prompt gamma-rays at 478 keV from ^7^Li (p, p′γ)^7^Li and 197 keV of ^19^F (p, p′γ)^19^F was carried out using a 30% HPGe detector. *In situ* current normalized count rate was used for concentration calculation by relative method. Details regarding the calculations can be found in ref. 29 and 30. As will also be reported later, the concentrations of lithium in the four samples were found to be in the range of 3.7–4.7 wt%, with the associated uncertainties being in the range of 0.4–0.6% in the form of standard deviations of triplicate sample analyses.

### 2.3. Investigation of the electrochemical performances

The electrochemical behavior of electrodes prepared from the as-synthesized LFP-based powder samples (mixed with 20% carbon black and 10% PVDF binder, tape casted on to Al foils with 1.5 mg of active mass loading) were investigated using standard CR2032 coin cells with lithium metal foil as the counter/reference electrode and 1 M LiPF_6_ in equimolar EC and DMC as the electrolyte. The galvanostatic charge/discharge cycles were performed at current density corresponding to C/2 within the cell voltage range of 2.2–4.2 V using Neware's battery charger. The electrochemical impedance spectroscopy was carried out using Novocontrol Alpha-A High Frequency Analyzer in the frequency range of 1 Hz to 1 MHz with 70 mV AC voltage at room temperature. The impedance spectra were analyzed using ZView 2.9b software. Cyclic voltammetry has been performed using Biologic potentiostat/galvanostat (BCS 810) within a voltage range of 2.5–4.2 V at scan rate of 0.1 mV s^−1^.

## 3. Results and discussion

### 3.1. Phase evolution, particle morphology and characterization of carbon coating

**Fig. 1:**
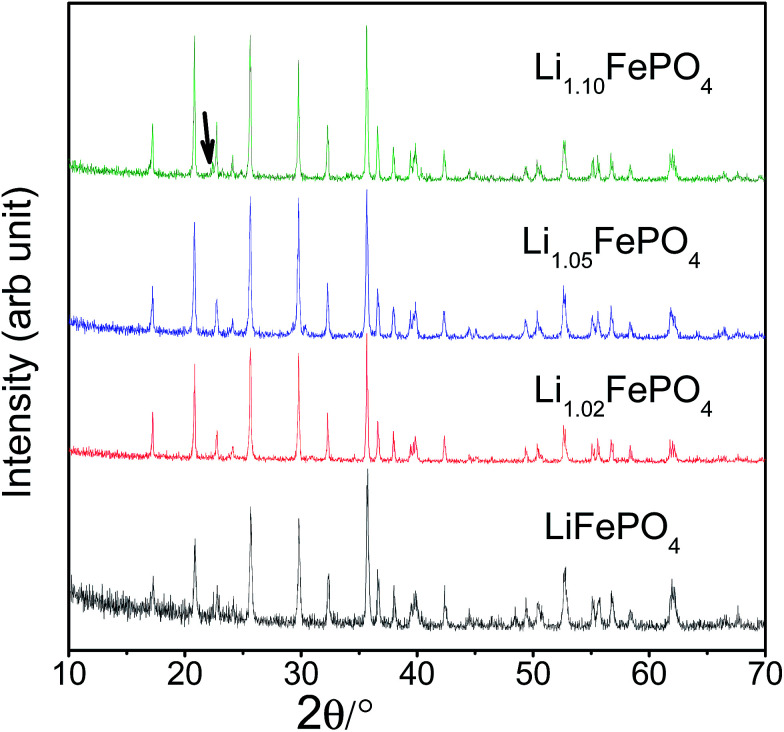
Representative XRD patterns recorded with all the four LFP-based samples (*i.e.*, A, B, C and D; or starting Li-contents corresponding to *x* = 1.0, 1.02, 1.05 and 1.10 in Li_*x*_FePO_4_).

XRD patterns recorded with all the Li_*x*_FePO_4_ (or LFP) based samples (*i.e.*, samples A to D) have been presented in Fig. 1. All the patterns look similar and the Bragg peaks can be indexed according to crystalline LiFePO_4_ phase possessing an ordered olivine structure with a *Pnma* space group (JCPDF file no: 40-1499). The crystallite sizes (*t*), as estimated based on the Scherrer's relation (*viz.*, *t* = 0.9*λ*/*β* cos *θ*, where *β* is the corrected full-width at half-maximum of the diffraction peaks and *λ* is the X-ray wavelength of 1.5406 Å) was found to be in the range 44–46 nm. The lattice parameters of the samples were determined using FullProf software and have been presented in Table 1. The minor peaks corresponding to Li_3_PO_4_ impurity phase could be observed at 2*θ* = 22.332° and 23.177° in sample D (as shown by arrow in Fig. 1). Accordingly, it seems LFP lattice cannot accommodate excess lithium and form solid solution, so lithium in excess precipitates in Li_3_PO_4_ form.^26^ Chen *et al.*^32^ had observed some other impurity phases like Li_4_P_2_O_7_, which, however, could not be detected here within the limit of powder XRD. In order to circumvent the limitation of XRD with respect to detecting the presence of impurity phases in very minute quantities and also in case some are amorphous in nature, Mossbauer spectroscopy has also been performed, which has been discussed later in Section 3.3.

**Table 1:** Refined lattice parameters of all the samples

Samples	*a* (Å)	*b* (Å)	*c* (Å)
Sample A	10.3224 (4)	6.0025 (3)	4.6857 (2)
Sample B	10.3233 (3)	6.0031 (2)	4.6881 (2)
Sample C	10.3232 (5)	6.0032 (3)	4.8951 (3)
Sample D	10.3243 (5)	6.0038 (3)	4.8889 (3)

**Fig. 2:**
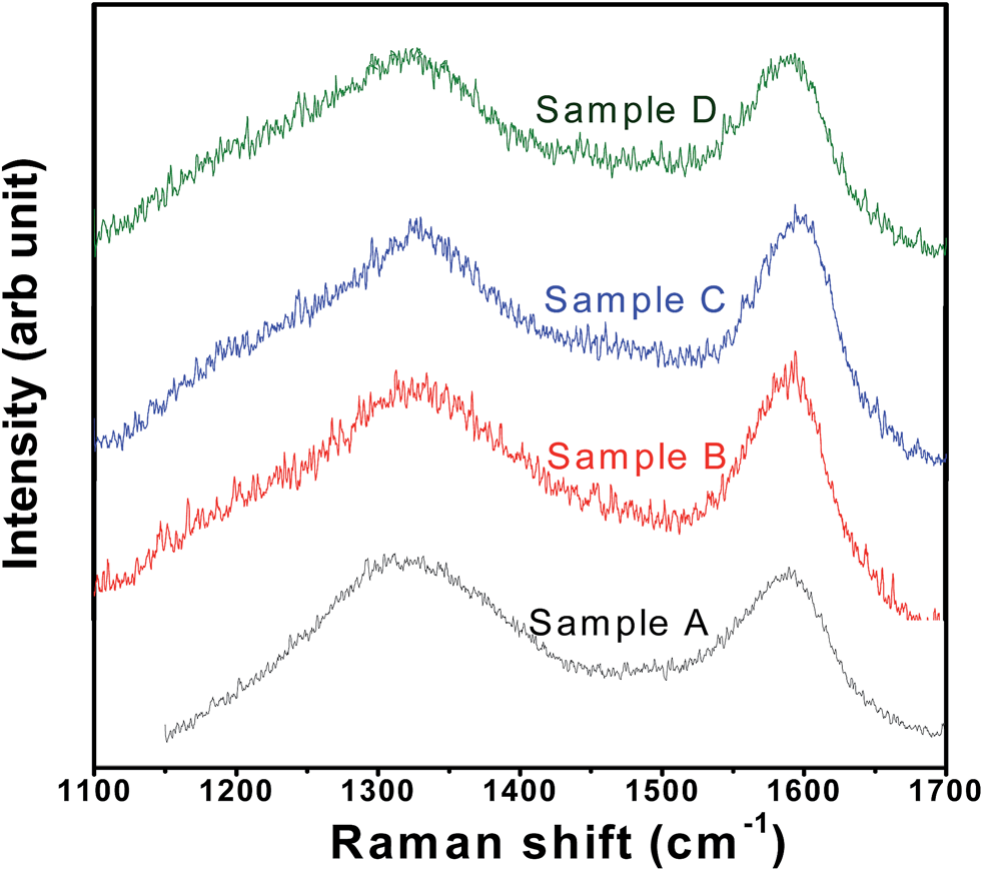
Typical Raman spectra for LFP-based samples (*i.e.*, samples A, B, C and D).

SEM images of the samples have been presented in ESI Fig. SI 1† which shows that the particles are of irregular shape. Mapping of Fe in all the samples have been performed to investigate the homogeneous distribution of Fe throughout the samples (see Fig. SI 2†). Raman spectra of all the samples show the presence of broad bands at 1358 (*i.e.*, D-band from A_1g_ vibration; partly representative of disorderness) and 1590 cm^−1^ (*i.e.*, G-band from graphitic E_2g_ vibration) (see Fig. 2). Not much variation in the *I*_D_/*I*_G_ ratios (*i.e.*, ∼1.02, ∼0.97, ∼0.97, ∼0.99, for samples A, B, C and D, respectively) could be seen across the sample sets, suggesting not much difference in the character (including graphitic nature and electronic conductivity) of the carbon-coatings.

### 3.2. Elemental analysis

**Fig. 3:**
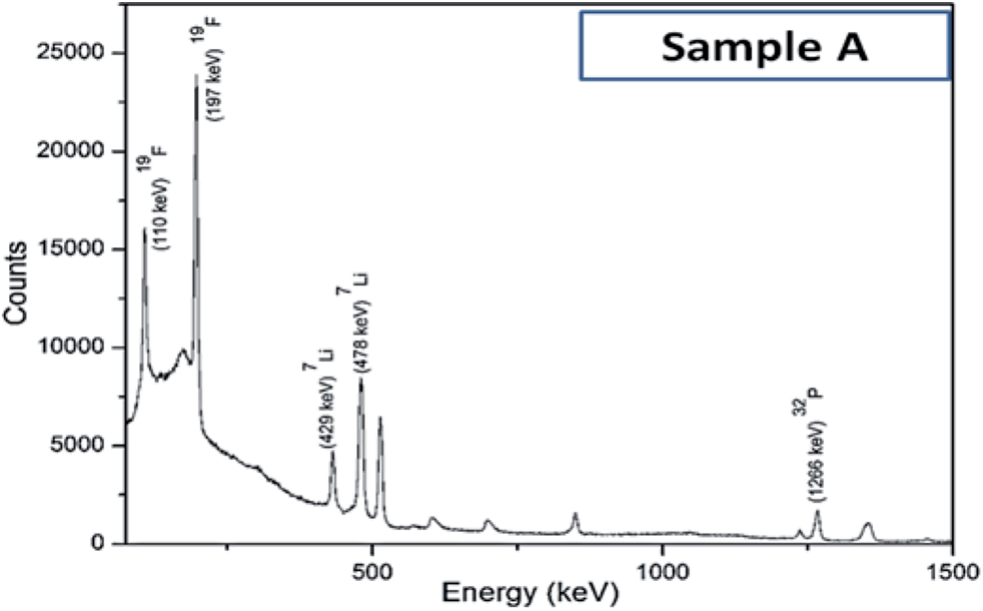
Typical gamma-ray spectrum of sample A, as obtained with proton induced gamma emission (PIGE) technique.

Iron concentration was determined by ICP-OES, while the lithium concentration was determined using the PIGE technique since it could not be accurately measured by either ICP-OES or AAS. A typical gamma ray spectrum of sample A has been presented in Fig. 3. Not surprisingly, due to Li volatilization primarily during the calcination step, the ratios of Li : Fe in the as-synthesized samples have been found to be lower compared to the nominal ratios at prior to synthesis; which in turn makes it mandatory to do the systematic analysis, as reported in the present work. It has also been reported earlier^19,28^ that due to such preferential evaporation of lithium, formation of lithium deficient compounds like FePO_4_, Fe_2_P_2_O_7_, FeP *etc.* are often encountered during synthesis. As a result, the actual proportion/amount of the electrochemically active LiFePO_4_ phase becomes less in the as-synthesized powders, which has been characterized here by Mossbauer spectroscopy. The as-obtained ratios Li : Fe for the four different sample types is given in Table 2. These tables enable to ascertain the amount of Li that gets preferentially lost during the synthesis, and accordingly provides a recipe to adjust the ratio of the precursors.

**Table 2:** Atomic ratios of Li : Fe in the as-synthesized samples, as determined by proton induced gamma emission (PIGE) technique (for Li) and ICP-OES (for iron). Error in determination of Li and Fe concentration was 0.4–0.6% and 5%, respectively

	Nominal composition	Li : Fe (atomic ratio)
Sample A	LiFePO_4_	0.96 : 1
Sample B	Li_1.02_FePO_4_	0.97 : 1
Sample C	Li_1.05_FePO_4_	1.02 : 1
Sample D	Li_1.1_FePO_4_	1.16 : 1

### 3.3. Mössbauer spectroscopy studies

Mössbauer spectroscopy is more sensitive than XRD for detecting iron based impurities and their chemical state because it involves direct analysis of electron density around iron ions. In any Mössbauer spectra, isomeric shift (IS) indicates the oxidation state of Fe in the compound and quadrupole splitting (QS) arises due to Coulomb field of the surrounding ligands. Accordingly, this spectroscopy has been used to develop further insights into the minute differences between the as-synthesized LFP-based samples in terms of their phase assemblage and composition, which are expected to have influence on the electrochemical performances (as will be reported in subsequent Section 3.4).

**Fig. 4:**
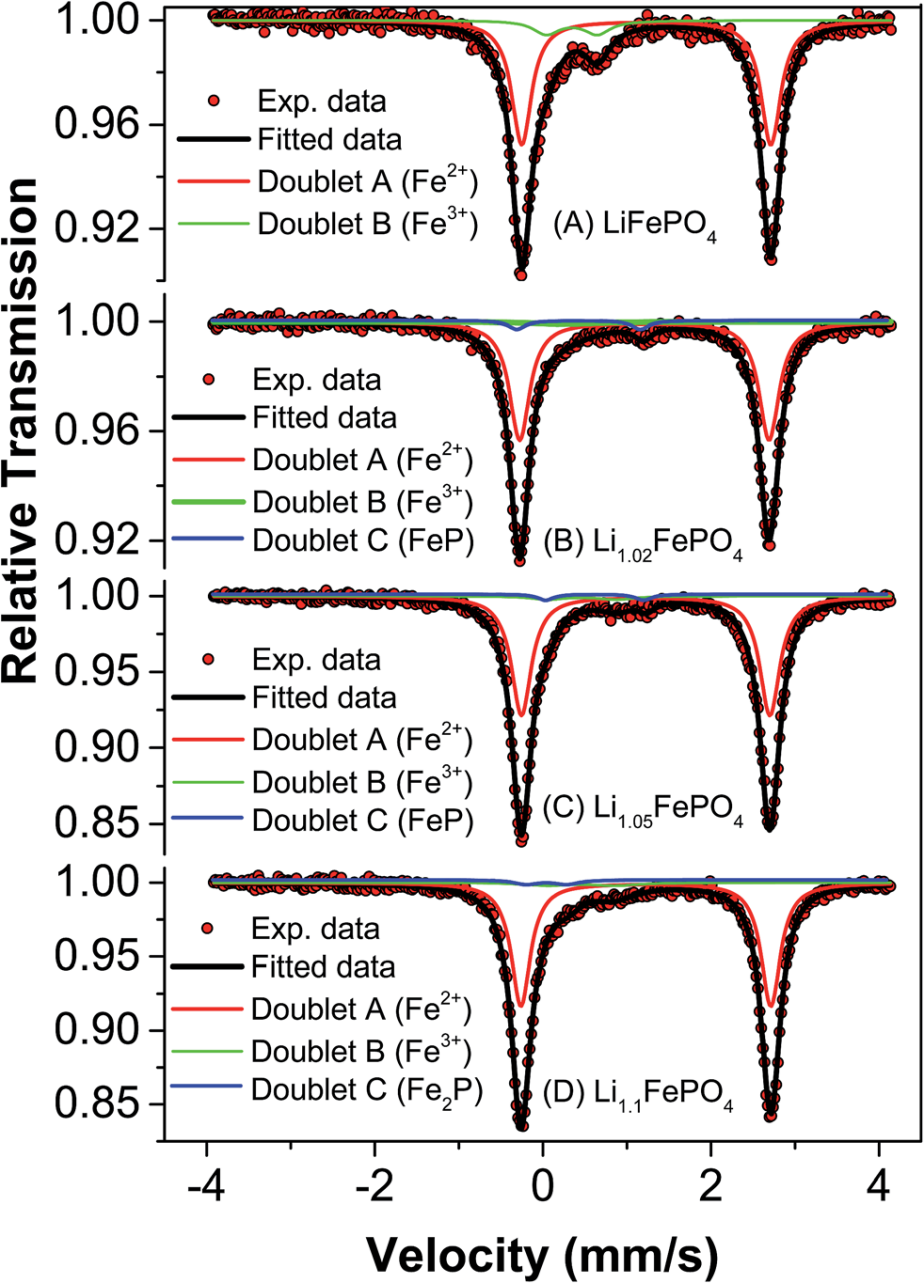
Mössbauer spectra of samples A, B, C and D.

Mössbauer spectra and parameters of all the samples have been presented in Fig. 4 and Table 3, respectively. The Mössbauer spectra of sample A could be well fitted into two doublets. The isomeric shift of the dominant doublet is found to be at 1.232 mm s^−1^ with quadrupole splitting (QS) as 2.964 mm s^−1^. This doublet corresponds to octahedral Fe^2+^ in ionic LiFePO_4_ and the relatively large QS is due to high spin configuration of d electron of Fe^2+^ (t^4^_2g_e^2^_g_) and asymmetric electronic arrangement. The another doublet with IS and QS as 0.345 mm s^−1^ and 0.606 mm s^−1^, respectively, could be assigned to Fe^3+^ at octahedral site with high spin state. Interestingly, these Mössbauer parameters do not match well with either FePO_4_ or Fe_2_P, which has otherwise been reported to form during the synthesis of LiFePO_4_.^19,33^ In the second sample (*i.e.*, sample B), three doublets have been observed which could be fitted well using previously reported data. In this sample, doublets corresponding to LiFePO_4_, FePO_4_ and FeP could be observed. More importantly, LiFePO_4_ concentration (88.2%) is found to be higher than that in the first sample (84.4%). Another striking observation is that the impurity FeP, which is known to be conducting, could be detected in this sample. Several authors have described its positive impact on electrochemical behaviour of LiFePO_4_.^33,34^ In the next sample (*i.e.*, sample C), it has been observed that the concentration of LiFePO_4_ further increases to 91.5%, but with Fe^3+^-based impurity still being present. The IS and QS of the Fe^3+^-based compound matches with that of Fe_4_P_6_O_21_. The conducting FeP phase could be observed in this sample also. In the fourth sample (*i.e.*, sample D) the concentration of LiFePO_4_ was found to be lower (86.9%) than the samples B and C.

**Table 3:** Mössbauer parameters of samples A (LiFePO_4_), B (Li_1.02_FePO_4_), C (Li_1.05_FePO_4_) and D (Li_1.1_FePO_4_)

Sample code	Fe sites	Quadrupole splitting (Δ *E*_Q_) mm s^−1^	Isomer shift (*δ*) mm s^−1^	Line width (*Γ*) mm s^−1^	Relative area, *R*_A_ (%)	Goodness of fit (*χ*^2^)
A	Doublet A (Fe^2+^) LiFePO_4_	2.964 ± 0.002	1.232 ± 0.001	0.287 ± 0.003	84.4	1.17213
	Doublet B (Fe^3+^)	0.606 ± 0.015	0.345 ± 0.009	0.373 ± 0.017	15.6	
B	Doublet A (Fe^2+^) LiFePO_4_	2.967 ± 0.003	1.209 ± 0.001	0.29 ± 0.001	88.2	1.1305
	Doublet B (Fe^3+^) FePO_4_	0.151 ± 0.06	0.238 ± 0.03	0.368 ± 0.1	7.4	
	Doublet C (FeP)	1.477 ± 0.08	0.457 ± 0.08	0.24 ± 0.1	4.4	
C	Doublet A (Fe^2+^) LiFePO_4_	2.97 ± 0.001	1.225 ± 0.003	0.266 ± 0.003	91.5	1.27856
	Doublet B (Fe^3+^) Fe_4_P_6_O_21_	0.769 ± 0.07	0.504 ± 0.03	0.451 ± 0.07	5.9	
	Doublet C (FeP)	1.171 ± 0.05	0.643 ± 0.02	0.23 ± 0.04	2.6	
D	Doublet A (Fe^2+^) LiFePO_4_	2.973 ± 0.001	1.227 ± 0.001	0.276 ± 0.002	86.9	1.0252
	Doublet B (Fe^3+^) Fe_4_P_6_O_21_	0.794 ± 0.07	0.516 ± 0.03	0.574 ± 0.1	8.0	
	Doublet C (Fe_2_P)	0.147 ± 0.05	0.458 ± 0.06	0.416 ± 0.09	5.1	

### 3.4. Electrochemical behaviour of the Li_*x*_FePO_4_-based samples

**Fig. 5:**
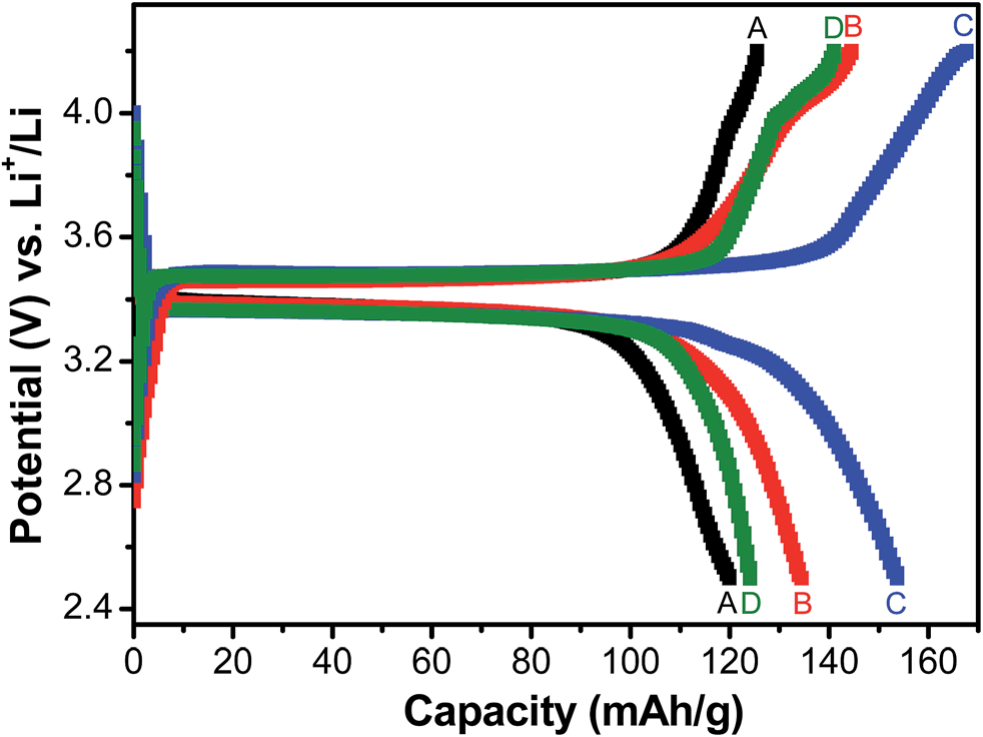
Galvanostatic charge–discharge behaviour of LFP-based samples A, B, C and D at current density equivalent to C/2 rate.

**Fig. 6:**
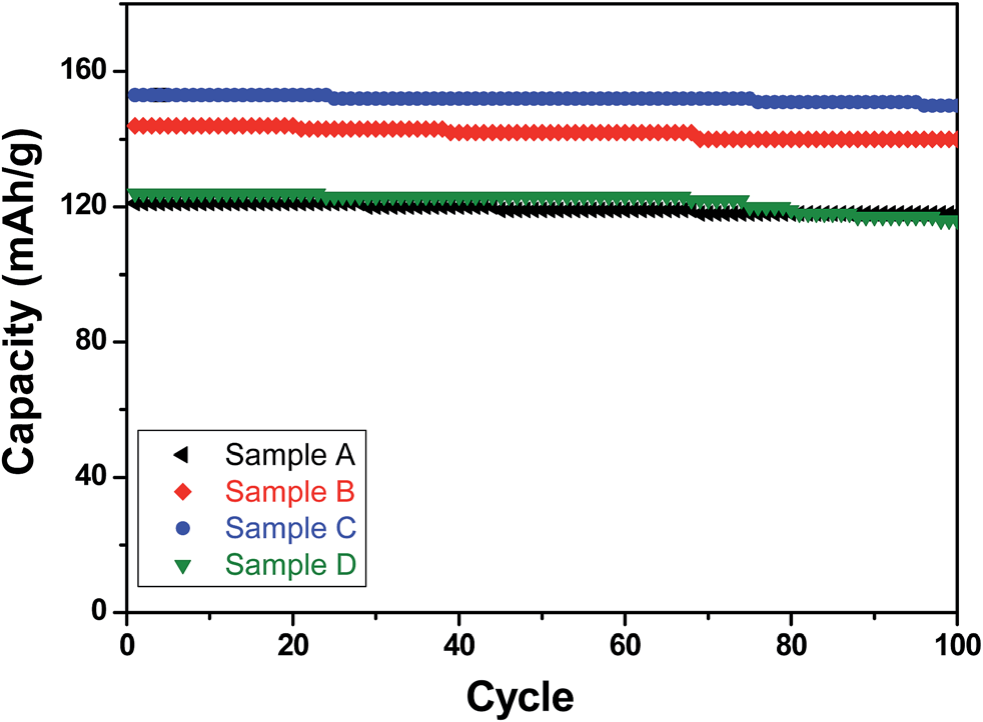
Specific (discharge) capacities recorded with the LFP-based samples A, B, C and D as functions of cycle numbers when galvanostatically cycled for up to 100 cycles at C/2 rate.

**Fig. 7:**
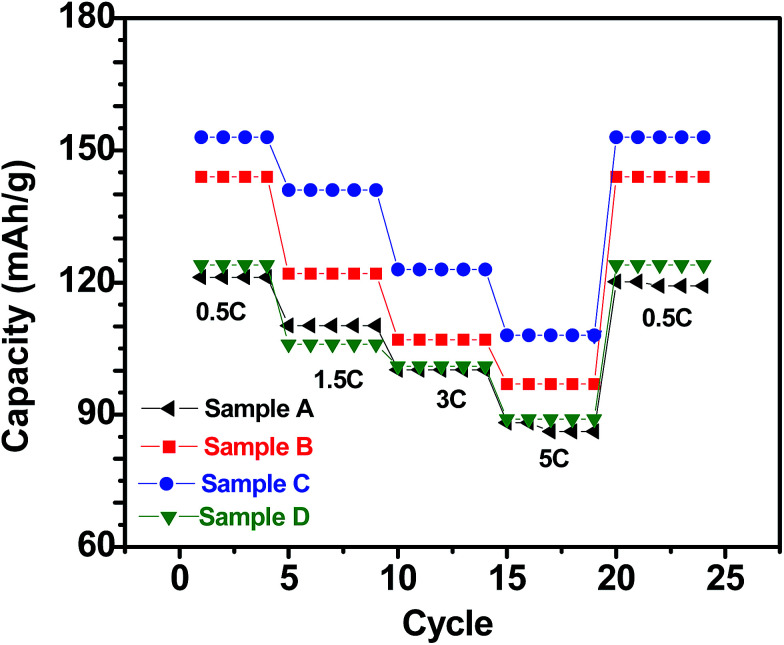
Specific (discharge) capacities recorded with the LFP-based samples A, B, C and D as at different current densities (C-rates).

Fig. 5 shows the charge/discharge profiles obtained with the electrodes based on samples A, B, C and D, when galvanostatically cycled at current density equivalent to C/2. The flat potential plateaus obtained at ∼3.4 V against Li/Li^+^ for all the samples indicate the occurrence of the usual reversible first-order phase inter-transformation between LiFePO_4_ and FePO_4_ during the electrochemical Li-removal/insertion. The coulombic efficiencies for the samples A, B, C and D are found to be ∼96%, ∼93%, ∼95% and ∼90%, respectively. More importantly, samples B and C have been observed to consistently possess greater capacity (by ∼25%, at C/2), as compared to samples A and D (see Fig. 5–7). It may be recalled here that samples B (Li : Fe ∼ 0.97) and C (Li : Fe ∼ 1.02) contained more Li that sample A (Li : Fe ∼ 0.96) (see Table 1). However, sample D had still greater Li-content (*viz.*, (Li : Fe ∼ 1.16)), but lower specific capacity. Additionally, insulating Li_3_PO_4_ has negative impact on capacity of sample D.^35,36^ This highlights the importance of optimization of Li content in as-synthesized Li_*x*_FePO_4_-based samples in very precise terms (*i.e.*, critical control of Li/Fe-precursor contents, based on thorough optimization, during synthesis). In order to develop more insights into the considerable effects noted in the electrochemical performance with such relatively minor variations in the Li : Fe ratios, it must also be recalled here that the contents of the actual electrochemically active phase, *i.e.*, LiFePO_4_, has been found to be ∼84.4%, ∼88.2%, ∼91.5% and ∼86% in samples A, B, C and D, respectively, as observed by Mossbauer spectroscopy (see Table 2), with the rest being the different Fe-based impurity phases. Based on this it is not surprising that sample C shows the highest specific capacity among all the samples. The LiFePO_4_ content of four samples was found to be 84.4%, 88.2%, 91.5% and 86.9% respectively. Capacities of these samples were found to be 122, 137, 150, 125 mA h g^−1^ when cycled at C/2 rate. It indicates that electrochemically active LiFePO_4_ could not be fully accessed for cycling. It has been calculated that only 71.7%, 80.5%, 88.2% and 73.5% of LiFePO_4_ of samples A, B, C and D, respectively, were accessible due to blocking by impurity phases or unsuitable morphology *etc.*

With respect to cyclic stability, the discharge capacity retentions after 100 galvanostatic cycles at C/2 for samples, A, B, C and D are ∼94%, ∼97%, ∼98% and ∼94%, respectively, of the corresponding first cycle capacities (Fig. 6). The slightly superior behaviour of samples B and C, as compared to A and D can be noted. In the context of rate capability, all the samples show the expected systematic decrease in specific capacity with increase in current density, with all the samples showing stable capacity retention in terms of recovering of the discharge capacity at C/2 rate after cycling through higher current densities (Fig. 7). More importantly, at a considerably high rate of 5C, the specific discharge capacities of samples B and C get retained at ∼99 and ∼107 mA h g^−1^, much better than those of samples A and D (∼85 mA h g^−1^). Overall, the results concerning electrochemical performances indicate that LFP-based samples B and C, especially C, are superior compared to samples A and D in all the aspects, *viz.*, specific capacity, cyclic stability and rate capability. It has been reported that formation of two major defects, namely 
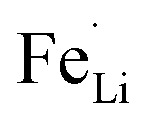
 and 
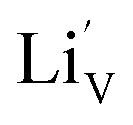
, (anti-site defect) are energetically favourable in off-stoichiometric LiFePO_4_ which also degrade the electrochemical properties severely.^37,38^ It is not unlikely that these might also have a role in our LFP-based samples with relatively inferior performances. The CV curves of all the samples have been presented in ESI Fig. SI 3†.

### 3.5. Electrochemical impedance spectroscopic (EIS)

**Fig. 8:**
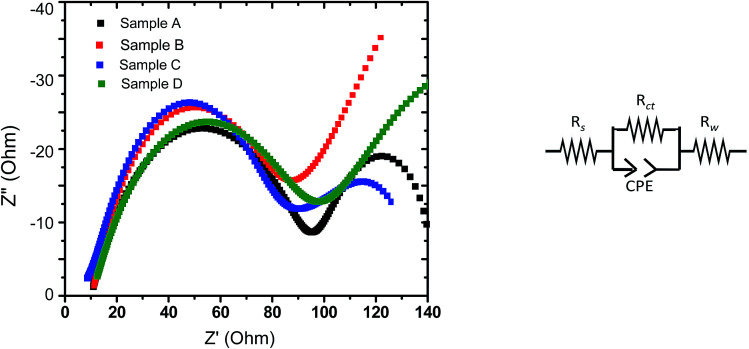
(Left panel) Nyquist plots obtained during EIS experiments with the LFP-based samples A, B, C and D (at discharged state). (Right panel) Circuit considered for fitting.

The complex impedance plots obtained with electrodes based on all the four samples (upon EIS experiments performed at discharged state) have been analyzed by fitting the curves to a model, as shown in Fig. 8. The parameters obtained based on such fits have been reported in Table 4, where *R*_s_ represents the electrolyte solution resistance, *R*_ct_ is based on the diameter of the semicircle at the higher frequency representing the charge transfer resistance and the inclined line corresponds to Warburg resistance (*R*_w_). A constant phase element (CPE) is introduced in the circuit to represent the double layer capacitance and the influence of passivation processes on electrode/electrolyte interface and other heterogeneities. As can be observed from Table 3, the charge transfer resistance of sample C is found to be the lowest among all the samples, followed by sample B. The exchange current, *i*_o_, can be derived from *R*_ct_ using following relation;

1 *i*_o_ = *RT*/*nFR*_ct_

where *n* is the charge transfer number per molecule during intercalation, *R* is gas constant (8.14 J mol^−1^T^−1^), *T* is temperature (298 K), *F* is Faraday constant (∼96 500 C mol^−1^). The values of *i*_o_, as estimated based on eqn (1), have been reported in the last column of Table 4.

**Table 4:** Solution resistance (*R*_s_), charge transfer resistance (*R*_ct_), Warburg resistance (*R*_w_) and exchange current estimated based on the EIS experiments performed with the LFP-based samples A, B, C and D electrodes based on samples A, B, C and D

Samples	*R*_s_ (Ohm)	*R*_ct_ (Ohm)	*R*_w_ (Ohm)	*i*_o_ (mA)
Sample A	10	76	11.0	0.335
Sample B	9	68	55.3	0.374
Sample C	9	57	54.9	0.446
Sample D	10	77	63.5	0.331

Accordingly, the EIS data indicate that part of the reasons behind the superior overall electrochemical performances (including rate capability) of the electrodes prepared from sample C is related to lower charge transfer resistance and accordingly the greater exchange current density (implying greater charge transfer kinetics). It may also be recalled here that the stoichiometry of sample C also led to the formation of a conducting impurity phase, *viz.*, FeP (as mentioned in Section 3.3). Such observations tend to indicate that the structural/interfacial defects/features and presence/absence of impurity phases caused due to off-stoichiometry (when deviate from the optimum Li concentration; as in samples A and D) negatively affected charge transfer resistance at the electrode/electrolyte interface (as opposed to the bulk transport), which in turn had the dominant effect on all the electrochemical performances.

## 4. Conclusions

Carbon coated Li_*x*_FePO_4_ based samples with systematically varied Li-contents were synthesized *via* a facile sol–gel route, followed by reduction under Ar : H_2_ atmosphere at 650 °C. XRD results confirmed the presence of crystalline LiFePO_4_ phase in all the samples. As determined in fairly precise terms using proton induced gamma emission (PIGE) technique (for Li) and ICP-OES (for Fe), the final Li : Fe ratios for the as-synthesized samples A, B, C and D were ∼0.96 : 1, ∼0.97 : 1, ∼1.02 : 1, ∼1.16 : 1, respectively confirming preferential lithium sublimation during calcination. ^57^Fe Mossbauer spectroscopy indicated that the composition/stoichiometry led to the presence of varied fractions of the actual electroactive phase (*viz.*, LiFePO_4_), with sample C having the highest content at ∼91.5%, followed by samples B (*i.e.*, ∼88.2%), D (*i.e.*, ∼86.9%) and A (*i.e.*, ∼84.4%). The other phases being primarily Fe-containing impurities, with FeP conducting impurity phase being also detected in samples B and C.

With respect to the electrochemical performances, the electrodes prepared from sample C show the best performance in all aspects (*viz.*, specific capacity, cyclic stability and rate capability). Furthermore, the electrode based on sample C could retain ∼107 mA h g^−1^ even when cycled at very high current density equivalent to 5C. Analysis of EIS data indicated that the electrode based on sample C possess the least charge transfer resistance, which in all probability partly accounted for the superior electrochemical behavior for the same. Overall, in more practical terms, the correlations between composition/stoichiometry, phase assemblage, impurity contents and electrochemical behavior, as obtained in the presently conducted systematic study, not only highlights the importance of precise control of the stoichiometry while synthesizing Li_*x*_FePO_4_-based electrode materials, but also throws insights into the desired Li : Fe atomic ratio for obtaining the best possible electrochemical performances.

## Conflicts of interest

There are no conflicts of interest.

## Supplementary Material

RA-008-C7RA10112K-s001
