# Supplementary material for: Optimization of lithium content in LiFePO4 for superior electrochemical performance: the role of impurities
Source: RSC Adv. 2018 Jan 3;8(2):1140–7. doi: 10.1039/c7ra10112k (PMC9076985; doi:10.1039/c7ra10112k)
Supplement: RA-008-C7RA10112K-s001 [file RA-008-C7RA10112K-s001.pdf]

Electronic Supplementary Material (ESI) for RSC Advances.
This journal is © The Royal Society of Chemistry 2017

Optimization of lithium content in $LiFePO_4$ for superior electrochemical performance: Role of impurities

Kruti K.Halankar[a], Balaji. P. Mandal[a,e*], Manoj K. Jangid[b], A. Mukhopadhyay[b], Sher S. Meena[c], R. Acharya[d,e], A. K. Tyagi[a,e*]

[a] Chemistry Division, Bhabha Atomic Research Centre, Mumbai – 400085, India
[b] High Temperature and Energy Materials Laboratory, Department of Metallurgical Engineering and Materials Science, IIT Bombay, Mumbai-400076, India
[c] Solid State Physics Division, Bhabha Atomic Research Centre, Mumbai – 400085, India
[d] Radiochemistry Division, Bhabha Atomic Research Centre, Mumbai – 400085, India
[e] Homi Bhabha National Institute, Mumbai – 400085, India

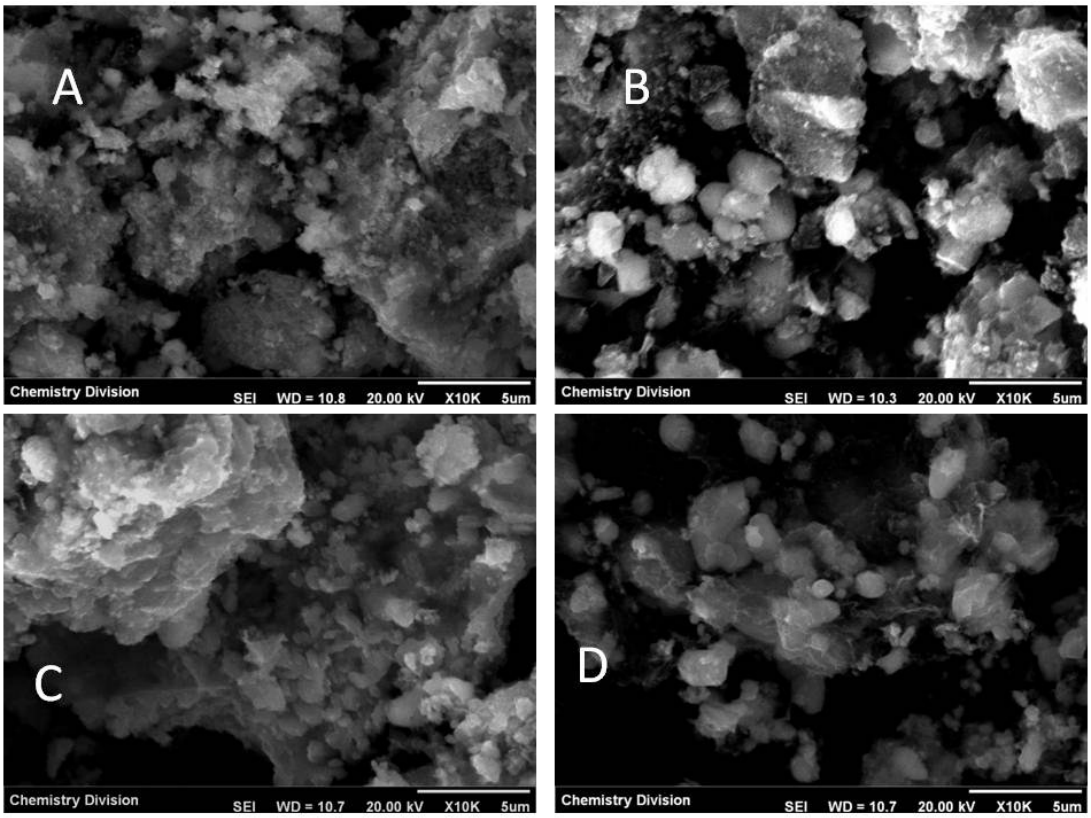

Fig. SI1. Representative SEM images obtained with (a) sample A, (b) sample B, (c) sample C and (d) sample D

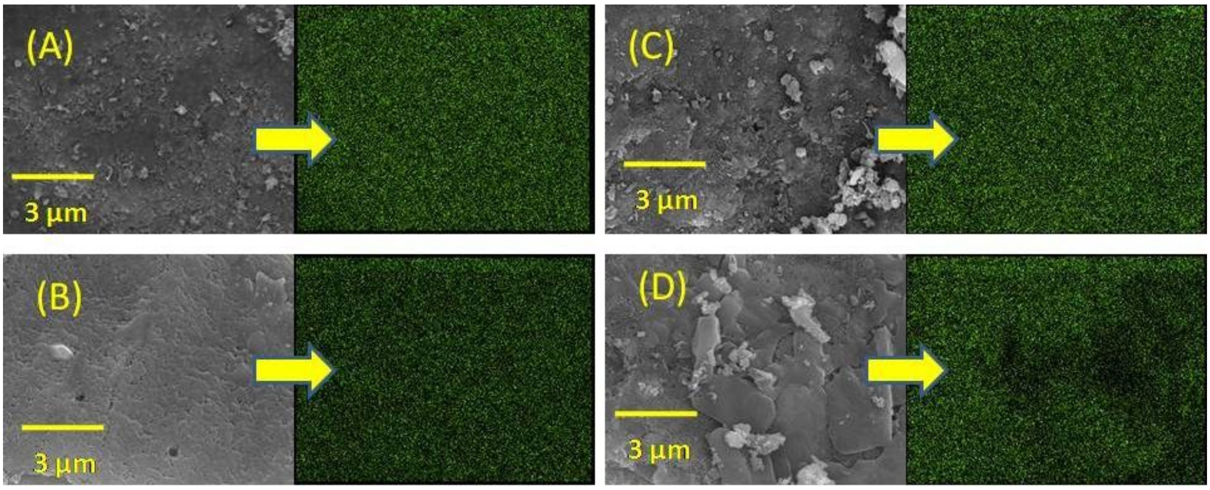

Fig. SI 2. Element maps of Fe as obtained in (a) sample A, (b) sample B, (c) sample C and (d) sample D by EDS.

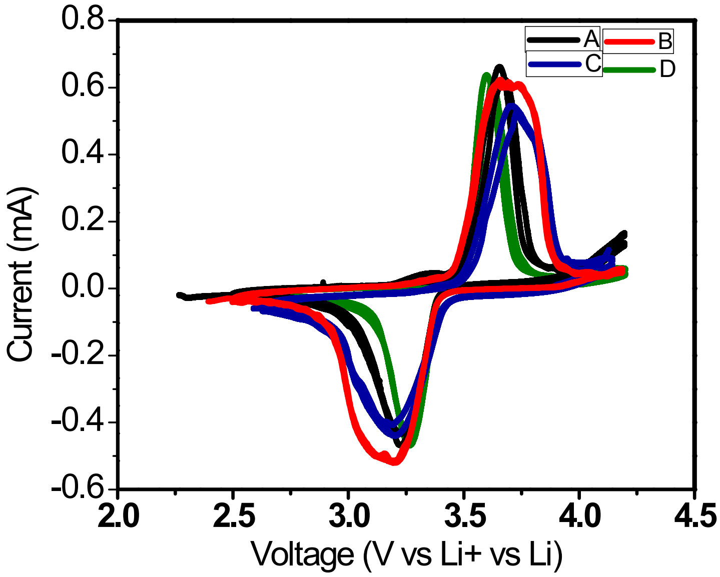

Fig. SI 3: CV plots of samples A, B, C and D.
